# Subungual Epidermoid Inclusions–A Series of 8 Cases and a Review of Literature

**DOI:** 10.1111/cup.14830

**Published:** 2025-06-11

**Authors:** Sarah M. Alnaqshabandi, Anurag Sharma, Ahmed Bakhshwin, Shira Ronen, Jennifer S. Ko, Steven D. Billings

**Affiliations:** ^1^ Pathology & Laboratory Medicine Institute Cleveland Clinic Cleveland Ohio USA; ^2^ Pathology & Laboratory Medicine, London Health Sciences Center Western University London Ontario Canada; ^3^ Department of Pathology King Abdulaziz University Jeddah Saudi Arabia

**Keywords:** epidermoid cyst, epidermoid inclusion, nail bed, onycholemmal cyst, subungual

## Abstract

Subungual epidermoid inclusions (SEI) are benign cystic lesions of the nail bed. To our knowledge, there has been only one case series describing SEI. We report eight cases of SEI. The patients had a median age of 72 years (range 3–84) with a female: male ratio of 1.6. Five occurred in toenails and three in fingernails. Histologically, SEI is characterized by bulbous proliferation of rete ridges and unilocular cysts lined by thin squamous epithelium with hypogranulosis, filled with orthokeratin. The connection to the nail bed epithelium may be disrupted and calcified. SEI are tumors that should be kept in the differential diagnosis of the subungual nail bed lesions.

## 1 Introduction

Subungual epidermoid inclusions (SEI) are benign cystic lesions of the nail bed, first described in the toenail in 1959 and later in the fingernail in 1965 [1, 2, 3]. The etiology remains unclear; however, two main hypotheses have been proposed, including previous trauma or surgery with entrapment of epidermal tissue and development from embryonic cell rests of the dermis and soft tissue [4]. There are two main types of SEIs “Intraosseous epidermoid cysts in the bones are painful and associated with osteolysis. Subungual extraosseous epidermoid cysts, also known as subungual epidermoid inclusion cysts, have variable clinical presentation such as subungual hyperkeratosis, ridging, clubbing, onycholysis, paronychia, pincer nail deformity, and onychodystrophy” [5, 6].

Clinical and histopathologic diagnosis of subungual epidermoid inclusions can be challenging. As such, a systematic approach, including physical examination, radiological imaging, and histopathological examination, is often needed to provide an accurate diagnosis and treatment to avoid permanent damage to the nail and maintain functionality [7].

This study aims to describe our experience with these eight cases, discuss the clinical, radiologic, and pathologic features of this entity, and review the literature.

## 2 Materials and Methods

Following approval by the Institutional Review Board, eight cases of SEI were identified (seven cases were from our institution and one consultation case) and retrieved from the dermatopathology files at our institution. The cases spanned a 15‐year period from 2008 to 2023. We reviewed 4‐μm‐thick hematoxylin–eosin‐stained sections from these cases. Clinicopathologic characteristics such as age, gender, location, histopathologic features, and clinical follow‐up data were recorded. A literature review was also performed, and a pooled data group of SEI cases reported from 1969 to 2021 was compared to the current group.

## 3 Results

### 3.1 Clinical Features

The clinical features for the cases in our series are summarized in Table 1. There were five females and three males (F: M = 1.6). The age range was 3–84 years (median = 72). Three cases involved the fingernails while the remaining five cases involved the toenails. A history of prior trauma was reported in four out of eight patients. The clinical signs were variable. Three out of eight cases reported local pain; nail discoloration was found in four out of eight cases; cutaneous horn, or wart‐like growth with nail thickening, was described in two out of eight cases; and nail dystrophy and ulceration were reported in one case each. The youngest patient, a 3‐year‐old with amniotic band syndrome, presented with hyperplasia of hands and feet (Table 2).

**TABLE 1:** Clinical characteristics of cases in this series.

Case	Sex	Age (years)	Location	History of trauma	Duration	Clinical features and diagnosis
1	M	3	Left index	(+)	N/A	Hyperplasia of hand and feet with degenerated nail. Amniotic band syndrome.
2	F	58	Right great toe	(−)	N/A	Pigmented lesion fungal infection
3	F	67	Right thumb	(+)	20 years	Painful nail. Wart
4	F	69	Right great toe	N/A	12 months	Thick black painful discoloration
5	M	75	Left third toe	(+)	N/A	Ulceration with bone necrosis and calcification, pain amputation
6	M	81	Right third finger	(−)	N/A	Erythronychia
7	F	82	Right great toe	(+)	N/A	Swelling of the toe, nail dystrophy
8	F	84	Right third toe	(−)	N/A	Cutaneous horn

Abbreviations: F, female; M, male; N/A, not available.

**TABLE 2:** Clinical features of published subungual epidermoid cysts: Review of the literature.

Authors	Sex	Age (years)	Location	History of trauma	Duration	Clinical features
Bukhari et al.	F	11	Left thumb	(−)	1 year	Growing lesion, mildly painful, transverse short grooving, ridging and loss of cuticle
Yung et al.	M	27	Left middle finger	(+)	3 years	Enlarged painful lesion. Subungual yellowish discoloration
Paulin et al.	M	30	Left third finger	(−)	N/A	Nail dystrophy
Bobra et al.	M	44	Right thumb	(+)	2.5 years	Thinning, destruction of the cortex with swelling of soft tissue
Molloy et al.	F	53	Left middle finger	(+)	2 weeks	Painful subungual lesion
Geisler et al.	M	54	Left thumb	(+)	1 year	Slowly growing lesion dystrophic nail plate chipping easily with whitish nodular lesion
Goktay et al.	F	60	Left thumb	(−)	6 months	Painless nail discoloration with line on the thumbnail
Takiyoshi et al.	F	63	Right thumb	(+)	2 months	Subungual pain Nail unremarkable
Kumar et al.	M	68	Bilateral big toes	(−)	10 years	Pain and intermittent purulent discharge, onycholysis, subungual hyperkeratosis and yellowish discoloration
Lewin et al.	M	71	Left thumb	(+)	1 year	Pain and swelling of paronychial folds. Black nodular swelling in nail bed
Fanti et al.	F	34	Left thumb	(+)	6 months	Onycholysis
	F	42	Toenail right and left	(−)	3 years	Subungual hyperkeratosis
	M	49	Right big toe	(+)	7 years	Marked subungual hyperkeratosis
	F	60	Right big toe	(−)	1 year	Subungual hyperkeratosis
	M	61	Left big toe	(+)	1 year	Nail plate thickening, subungual hyperkeratosis
	F	67	Right thumb	(+)	1 year	Nail plate thickening Subungual hyperkeratosis
	F	67	Right thumb	(+)	1 year	Nail plate thickening Subungual hyperkeratosis
	M	74	Fingernail	(−)	Several years	Subungual hyperkeratosis

Abbreviations: F, female; M, male; N/A, not available.

The duration of the nail abnormalities ranged from 7 months up to 20 years. All the cases involved a single digit. Co‐existing onychomycosis was present in one out of eight cases.

### 3.2 Radiologic Findings

Three out of eight cases (cases 1, 6, and 8) had radiographs performed prior to the biopsy, and one case (case 5) had a post‐surgical radiograph. Imaging from adult cases demonstrated degenerative changes of the interphalangeal joints with osteoarthritis and soft tissue calcification. The one pediatric case in our study (amniotic band syndrome involving multiple bilateral digits) demonstrated normal metacarpal and proximal phalanxes with truncation of the 2nd, 4th, and 5th middle phalanxes and amputation of the distal 2nd, 4th, and 5th distal phalanxes. The case with post‐surgical imaging showed changes of resection arthroplasty at the proximal interphalangeal joint of the 5th toe with mild to moderate degenerative changes at multiple interphalangeal joints. The joint spaces and alignment were normal with no fractures and normal soft tissue.

### 3.3 Histopathologic Features

**FIGURE 1:**
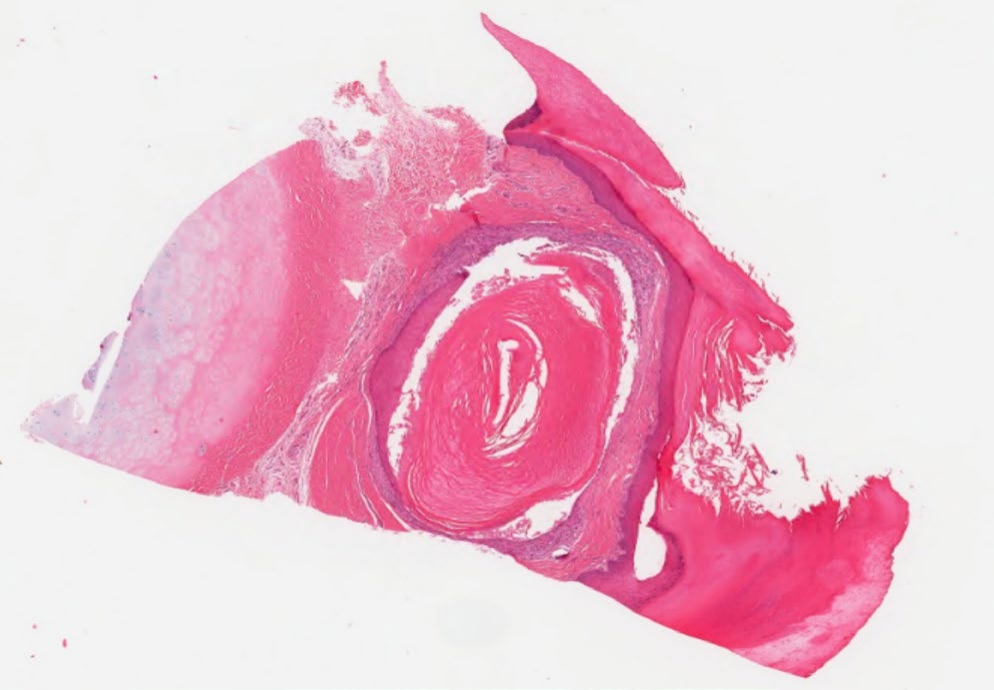
Unilocular cyst lined by epidermal‐type squamous epithelium with partially retained granular layer (H&E 20×).

**FIGURE 2:**
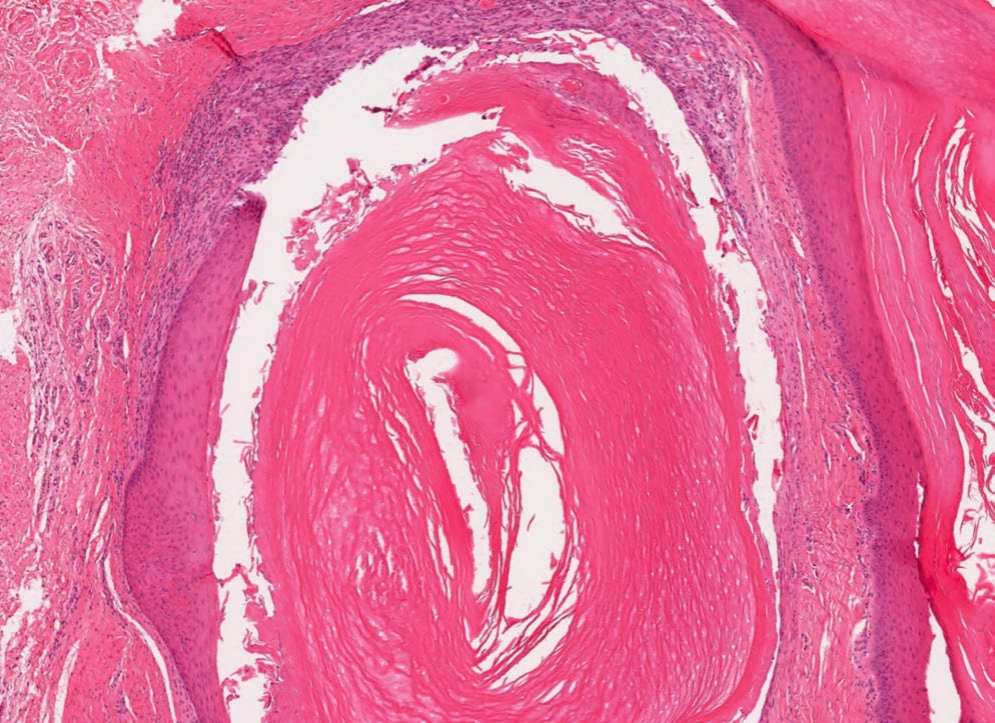
Unilocular cyst lined by epidermal‐type squamous epithelium (H&E, 40×).

**FIGURE 3:**
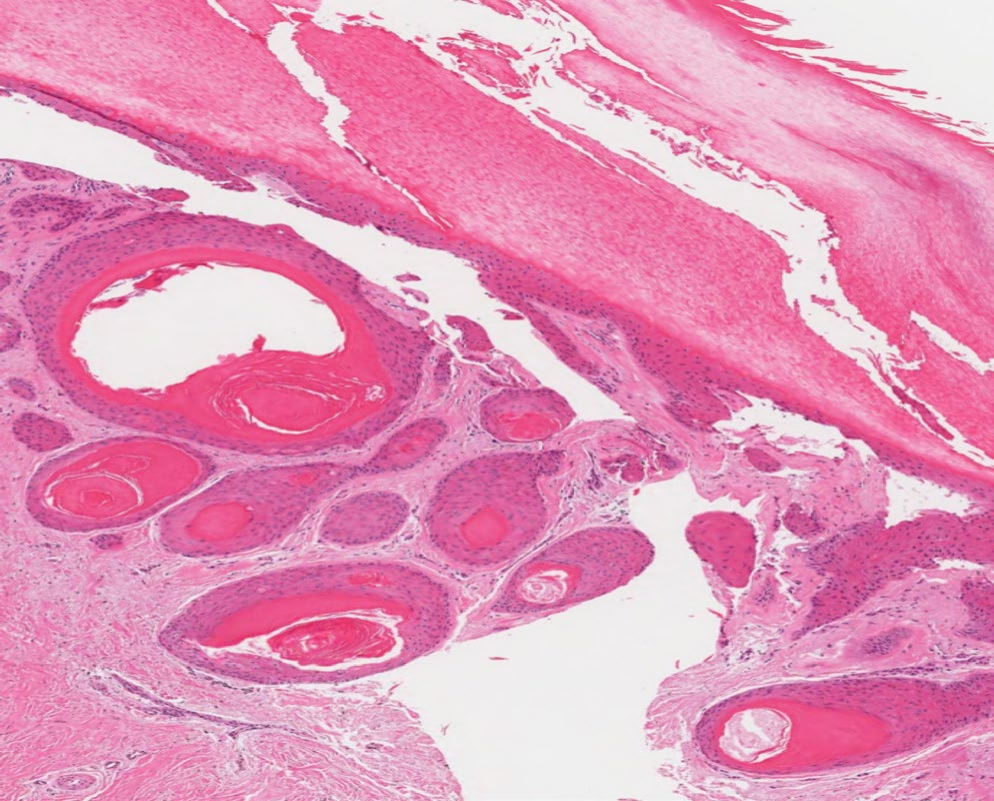
Keratin strands present within the cystic cavities (H&E, 40×).

**FIGURE 4:**
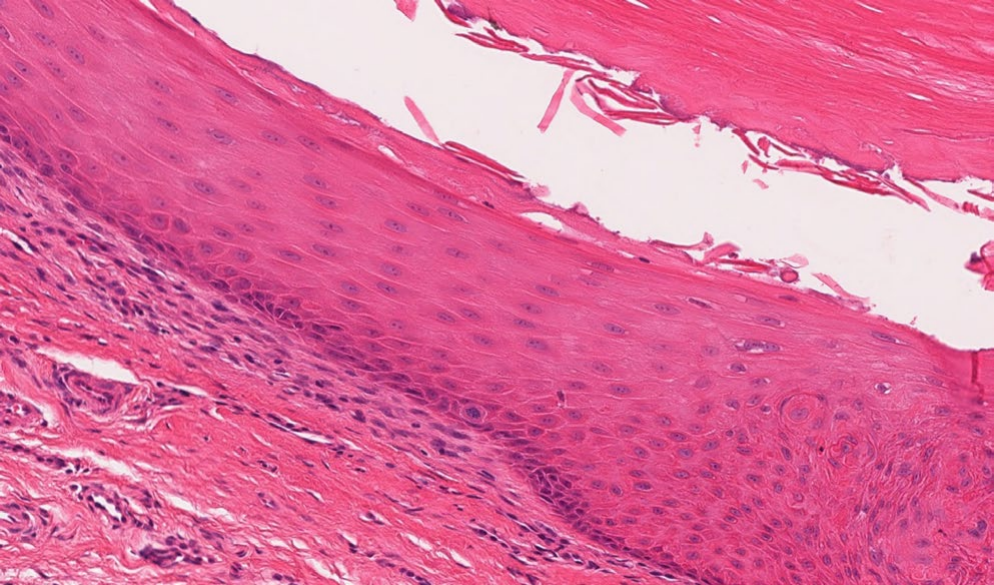
The keratinocytes exhibited light pink cytoplasm with ovoid, uniform nuclei (H&E, 100×).

The histopathologic features were similar in all cases. They revealed small cystic cavities lined by epidermal‐type squamous epithelium with a complete or interrupted granular layer (Figures 1 and 2). Keratin strands were present within the cystic cavities (Figure 3). The keratinocytes exhibited light pink cytoplasm with ovoid, uniform nuclei (Figure 4). Cytologic atypia, mitotic activity, or necrosis was not identified.

## 4 Discussion

Subungual epidermoid inclusions (SEI) have been described in the literature with only a few case reports and series, which are summarized in Table 2 [5, 8, 9, 10, 11, 12, 13, 14, 15]. Given the relatively sparse literature on SEI and the fact that the topic is not always covered in dermatopathology texts, it is our experience that dermatopathologists may be unfamiliar with the entity. To our knowledge, this represents one of the largest single series of SEI reported.

The prior series from 1969 was a retrospective study performed on nail bed samples collected from 90 autopsies to study nail abnormalities linked to different diseases. SEI were present in 8 cases (8.8%) who clinically had clubbing of fingers, but they were not found in association with any other abnormality [8]. No previous local trauma, nail disorders, or prior skin conditions were documented, and SEI were also observed in normal nails [8, 9].

The clinical features of subungual epidermoid inclusions (SEI) in our study are consistent with prior reports, with some notable variations. The age range of our patients was 3–84 years (median 72 years), slightly higher than the previously reported median age of 54 years (range 11–74) [5, 8, 9, 10, 11, 12, 13, 14, 15]. Our cohort showed a slight female predominance (ratio 1.6) compared to the pooled data group with a ratio of 1.1 [5, 8, 9, 10, 11, 12, 13, 14, 15]. A history of trauma was documented in four of eight cases in our series, aligning with 11 of 18 cases in the pooled data, suggesting trauma as a frequent, though non‐obligatory, contributing factor [5, 8, 9, 10, 11, 12, 13, 14, 15]. SEI in our study more commonly involved toenails (*n* = 5) than fingernails (*n* = 3), whereas pooled data indicated a predilection for fingernails (*n* = 13) over toenails (*n* = 5) [5, 8, 9, 10, 11, 12, 13, 14, 15], with a single case reporting bilateral involvement [10]. In all cases, including our study, involvement was limited to a single digit.

Clinical presentations were highly variable, with most patients being asymptomatic. When symptoms were present, they included nail discoloration, dystrophy, thickening, and, occasionally, a gradually increasing swelling of the distal phalanx, most commonly in the thumb or great toe [5, 8, 9, 10, 11, 12, 13, 14, 15]. Pain was less frequently observed in pooled cases, reported in only one case compared to three in our series [8, 9, 10, 11, 12, 13, 14, 15], alongside findings such as clubbing, onycholysis, ridging, paronychia, and onychodystrophy [6, 10]. Notably, an “eclipse sign” visualized by dermoscopy has been described as a characteristic feature of SEI [11]. Nail plate thickening and subungual hyperkeratosis were the most common findings in pooled data (seven of 18 cases), followed by onycholysis (two cases), soft cystic masses (three cases), and nail discoloration or dystrophy (six cases) [5, 8, 9, 10, 11, 12, 13, 14, 15]. Overall, while clinical presentations are diverse, nail changes such as discoloration, dystrophy, and thickening remain the most common findings, with multiple or bilateral SEI being exceptionally rare [10].

When employed, non‐invasive techniques such as radiological imaging may help shorten the list of differential diagnoses of nail bed lesions. The use of ultrasonography and color Doppler is useful to rule out vascularity, and the use of magnetic resonance imaging (MRI) excludes bone involvement [7]. Four out of eight cases (three pre‐surgical and one post‐surgical) in our study had an x‐ray performed. In the pooled data group cases, three had x‐rays and three had MRI. None of the radiographic imaging demonstrated bone involvement, soft tissue swelling, or vascular abnormalities.

The clinical differential diagnostic considerations include digital myxoid cyst, glomus tumor, hemangioma, subungual exostosis, soft tissue chondroma, keratoacanthoma, squamous cell carcinoma, and melanoma [7]. Histopathologic examination easily resolves most of these possibilities.

Histopathology of SEI is characterized by bulbous proliferation of rete ridges, a unilocular cyst lined by a thin epidermis with hypogranulosis, filled with orthokeratin. The connection to the nail bed epithelium may be disrupted, and calcification may be seen [3, 6]. Subungual epidermoid inclusions, as used in our manuscript, refer to asymptomatic, benign epithelial‐lined keratin‐filled structures without definitive evidence of trauma‐induced implantation. This terminology most accurately reflects the histopathological findings in our series and helps distinguish these lesions from implantation‐type epidermal inclusion cysts. All our cases had findings similar to the pooled data.

The primary histopathological differential diagnoses of SEI include onycholemmal cysts, onychomatricoma, subungual squamous cell carcinoma, and subungual keratoacanthoma. The clinically indistinguishable onycholemmal cysts are isthmus catagen cysts that can be found in the nail bed of the fingers or toes. Histologically, these cysts are lined by a thin epidermis with concentric keratin and lack a granular layer, which helps differentiate this entity from SEI [12, 13, 14, 15].

Onychomatricoma is a rare benign slowly growing nail matrix tumor [16]. Clinically, onychomatricoma is usually reported in middle‐aged females, presented with a thickened yellowish nail plate, transverse over the curvature of the nail plate, small cavities of the distal nail plate, and splinter hemorrhage [17]. The use of dermoscopy can be of benefit as it shows perforation in the distal portion of the nail plate, hemorrhagic striae, and white longitudinal lines representing the nail plate channels [18]. Histopathology, the features of onychomatricoma are distinctive. It is a biphasic fibroepithelial tumor originating from the nail matrix. The proximal part of the tumor is located under the posterior nail fold, showing deep epithelial invaginations covered by matrix epithelium like the normal nail matrix and causing nail thickening distally. The distal part of the tumor, which is associated with the lunula, shows multiple small cavities that perforate the nail plate and are filled with serous fluid [19].

The most important histopathologic differential diagnoses include subungual squamous cell carcinoma (SCC) and keratoacanthoma. Subungual SCC is the most common primary malignant neoplasm of the nail bed [20]. Typically, subungual SCC affects middle‐aged individuals (50–59 years), and it is often present as a persistent subungual lesion that primarily affects the thumb [21]. Histopathology, subungual SCC is more cellular, with nuclear atypia, mitotic activity, and an infiltrative border, features not seen in SEI [22].

Subungual keratoacanthoma affects men and is more likely to occur on the first three fingers of the hand, especially the thumb [23]. It is characterized by a painful, rapidly growing localized nodular lesion with a central keratin‐filled crater. The diagnosis is based on the correlation of clinical, radiological, and histological findings. Although histopathology is like other solitary keratoacanthomas, characterized by proliferative lobules of squamous epithelium composed of large keratinocytes with glassy eosinophilic cytoplasm, the subungual type exhibits more pronounced dyskeratosis with little or no nuclear atypia, patchy lymphocytes, and plasma cells infiltrates with little or no fibrosis at the base of the lesion [24]. SEI lacks dyskeratosis, has smaller keratinocytes, and typically lacks the associated inflammatory infiltrate.

Surgery is the treatment of choice for SEI, which is also performed to confirm the diagnosis [2].

In conclusion, SEI are benign lesions and should be kept in the differential diagnosis of the subungual nail bed lesions, especially in a post‐traumatic setting. Careful attention to histopathologic features allows accurate diagnosis.

## Ethics Statement

This case series was conducted following approval by the Institutional Review Board of the Cleveland Clinic Foundation (IRB07‐576).

## Conflicts of Interest

The authors declare no conflicts of interest.

## Data Availability

Data available on the request from the authors: The data that support the findings of this study are available from the corresponding author upon reasonable request.
